# The Norwegian healthy body image programme: study protocol for a randomized controlled school-based intervention to promote positive body image and prevent disordered eating among Norwegian high school students

**DOI:** 10.1186/s40359-018-0221-8

**Published:** 2018-03-06

**Authors:** Christine Sundgot-Borgen, Solfrid Bratland-Sanda, Kethe M. E. Engen, Gunn Pettersen, Oddgeir Friborg, Monica Klungland Torstveit, Elin Kolle, Niva Piran, Jorunn Sundgot-Borgen, Jan H. Rosenvinge

**Affiliations:** 1 Department of Sports Medicine, The Norwegian School of Sport Sciences, P.O. Box 4014, Sognsveien 220, N-0806 Oslo, Norway; 2 Department of Sports, Physical Education and Outdoor Studies, University College of Southeast Norway, P.O. Box 235, N- 3603 Kongsberg, Norway; 3 Faculty of Health Sciences Department of Health and Caring Sciences, UiT -The Arctic University of Norway, N- 9037 Tromsø, Norway; 4 Faculty of Health Sciences Department of Psychology, UiT –The Arctic University of Norway, 9037 Tromsø, Norway; 5 Faculty of Health and Sport Sciences, University of Agder, P.O. Box 422, 4604 Kristiansand, Norway; 6 Department of Applied Psychology and Human Development, University of Toronto, 252 Bloor Street West, Toronto, ON M5S 1V6 Canada

**Keywords:** Health promotion, Disease prevention, Body image, RCT-protocol, Adolescents

## Abstract

**Background:**

Body dissatisfaction and disordered eating raise the risk for eating disorders. In the prevention of eating disorders, many programmes have proved partly successful in using cognitive techniques to combat such risk factors. However, specific strategies to actively promote a positive body image are rarely used. The present paper outlines a protocol for a programme integrating the promotion of a positive body image and the prevention of disordered eating.

**Methods and design:**

Using a cluster randomized controlled mixed methods design, 30 high schools and 2481 12th grade students were allocated to the *Healthy Body Image* programme or to a control condition. The intervention comprised three workshops, each of 90 min with the main themes *body image*, *media literacy*, and *lifestyle*. The intervention was interactive in nature, and were led by trained scientists. The outcome measures include standardized instruments administered pre-post intervention, and at 3 and 12 months follow-ups, respectively. Survey data cover feasibility and implementation issues. Qualitative interviews covers experiential data about students’ benefits and satisfaction with the programme.

**Discussion:**

The present study is one of the first in the body image and disordered eating literature that integrates a health promotion and a disease prevention approach, as well as integrating standardized outcome measures and experiential findings. Along with mediator and moderator analyses it is expected that the *Healthy Body Image* programme may prove its efficacy. If so, plans are made with respect to further dissemination as well as communicating the findings to regional and national decision makers in the education and health care services.

**Trial registration:**

The study was registered and released at ClinicalTrials.gov 21th August 2016 with the Clinical Trial.gov ID: PRSNCT02901457. In addition, the study is approved by the Regional Committee for Medical and Health Research Ethics.

## Background

Body dissatisfaction (BD) is reported by up to one-third and every other adolescent boy and girl, respectively [1–4]. Quantitative studies have found that marked BD clusters with physical inactivity and weight gain [5–8] lower self-esteem [9], depressed mood [10, 11], social anxiety [12], perfectionistic concerns [13], and disordered eating (DE) [14]. Notably, across studies BD and DE are consistent risk factors for eating disorders (ED) [15], and it has been shown that both BD and perfectionistic concerns moderate high levels of ED symptoms [16]. A number of prevention programmes to combat BD and DE have been developed and tested during the past decades as indicated in reviews and meta-analyses [17–21].

These prevention programmes can be classified along two dimensions. The *first* dimension relates to target *populations*, and may be divided into a universal, indicative, and selective level [22]. The universal level targets the general population or specific demographic strata herein. Public schools have been the preferred arena for implementation of many ED prevention programmes due to high accessibility to adolescents, who are in a learning environment, and at the same time exposed to many risk factors [18, 19, 23]. Prevention programmes at the second (indicative) and third (selective) level addresses only individuals with known risk factors for a given disease, and individuals actually having a particular disease, respectively.

The *second* dimension is related to the *programme content and focus*. In many programmes, a universal approach and a health promotion perspective overlap. Given the prevalence of risk factors for EDs in the general population, notably BD [1–4], universal prevention programmes may also take an indicative approach. Within a disease prevention paradigm, the success of a programme hinges on whether the prevalence of one or more risk factors is reduced, and ultimately, whether the incidence of clinical cases is reduced.

Largely within a disease prevention paradigm several reviews and meta-analyses [15, 17, 20] indicate many beneficial outcomes of programmes targeting BD and DE. In the meta-analysis by Stice et al. [20] 51% of the included programmes were effective in reducing ED risk factors. Moreover, larger effects were found for multisession programmes using a selected (females 15 years or older, and at risk for ED) rather than a universal strategy for programmes targeting risk factors by persuasion approaches, notably cognitive dissonance techniques, compared to programmes with a pure psychoeducational approach. A more disturbing finding was the decline in effect sizes over time. A subsequent meta-analysis [17] found that approaches to increase media literacy to fight internalization of unhealthy body ideals were the only universal interventions that had small to moderate effect sizes of reducing risk factors. Although the methodology in previous studies have improved over the decades, many studies suffer from limitations like low statistical power [24], lack of long term follow-up [25], and a failure to use standardized measures of positive body image (and not just BD) [26] suitable for both genders [20, 27–29]. A possible floor effect of studying variables with a pathological twist within a relatively healthy population may account for modest effect sizes. In addition, less is known about the feasibility of interventions and experiential data from programme participants about possible programme benefits. Such limitations set standards for future research.

By contrast, a health promotion paradigm focuses on promoting general mental (or physical) health. It has been argued [30, 31] that the presence of a positive body image is not just the negation of a negative body image represented as BD and that at best, a neutral body image is the result of a disease prevention strategy [3, 31]. Hence, a disease prevention perspective may miss several aspects of a positive body image [32–34]. Qualitative studies [31, 32] indicate that a positive body image is multifaceted, including body appreciation [35], embodiment [33], a focus on body functionality rather than physical appearance and attraction as well as self-compassion [36] and acceptance of imperfection. Still, there are some overlap in the sense that a partial or contextually related BD may exist despite an overarching and inner sense of body appreciation [30].^.^

Reviewing mainly health promotion programmes [37] has revealed overall small to medium effect sizes for studies focusing on media literacy, self-esteem and the influence of peers. More recent studies indicate that actively promoting a positive body image increases physical activity level, decreases DE, dieting, alcohol consumption and cigarette use [38, 39] and that a mindful, non-judgmental and compassionate attitude to one’s body may protect against self-objectification and a negative body image [40]. Such positive outcomes may then contribute to resiliency towards unhealthy sociocultural body ideals.

Research on how to promote a positive body image may be essential to the future of prevention of DE and ED [3]. Acknowledging the high prevalence of BD [1, 4], it is suggested [34, 41, 42] that prevention programmes in general should encompass both a disease prevention perspective, i.e. targeting and reducing the prevalence of risk factors, as well as a health promotion perspective. Apart from one study [43] joint focus on alleviating BD and reducing DE, as well as promoting a positive body image has been scarcely focused. Therefore, integrating health promotion and disease prevention is the rationale for the development of the Norwegian Healthy Body Image (HBI) programme. The primary outcome measures are to promote a positive body image and to prevent DE. The purpose of the present paper is to outline the HBI-protocol in terms of the programme content, the study design, the procedures for randomization, recruitment and data collection in order to evaluate the immediate and long-term programme efficacy. Publishing the protocol may address the plea to avoid duplicate efforts, and to aspire for coordinated and strategic approaches needed to increase knowledge about effective school-based body image interventions [21].

### Aims and research questions

The overall aim of the study is to promote a positive body image, and to prevent DE among adolescents. The following research questions are addressed:

- Do participants in the HBI programme display a more positive body image compared with control students?
- Do participants in the HBI programme display less DE compared with control students?
- Will participants in the HBI programme adopt a healthier lifestyle compared with control students?
- What is the role of mediator and moderator variables?
- How do local programme administrators evaluate the programme feasibility?
- How do the students experience participating in the programme?

## Design and methods

This study has a mixed method design in which both quantitative and qualitative methods will be applied for data collection. Following the procedure of a randomized controlled study [44] the participants have been allocated to either the HBI programme or a control condition.

Standardized instruments will be used to measure programme efficacy. Understanding the determinants of intervention success or failure, and insight into the nature of the intervention delivery is essential. Therefore, we will perform an evaluation among participating students as well as local programme administrators. The administrators will respond to predefined questions about the feasibility of procedures. A selection of students will be invited to individual, semi-structured interviews. The selection will be made to accomplish maximum variation in experiences from participating in the programme.

**Fig. 1:**
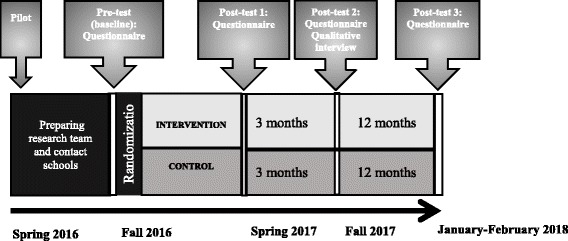
Study flow of the HBI program

A 1:1 ratio for cluster-randomization was conducted by a professional not affiliated with the project team to minimize contamination biases within schools. Schools were the selection units to avoid spillover effects due to communication about the intervention between participants and controls within each school. Figure 1 provides an overview of the study flow and the data collection intervals. During the intervention period students at the control schools continued following their regular school curriculum.

### Recruitment

**Fig. 2:**
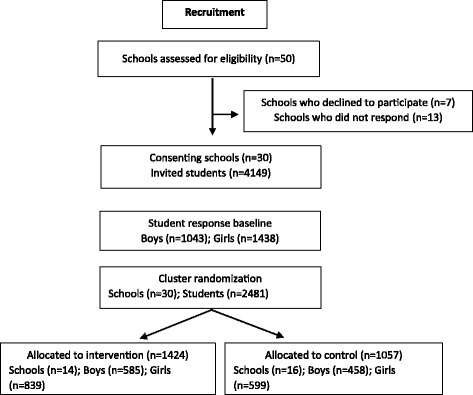
Recruitment and cluster randomization of participants

Following the recruitment procedure (Fig. 2) 30 schools and 2481 students were finally included.

The HBI programme includes 12th grade high school classes with both genders and with no exclusion criteria. All principals at every public and private high schools in Oslo and Akershus County in Norway were contacted during May–September 2016. At the consenting schools, detailed study information was provided to students and staff. After signing a letter of consent through e-mail, students were given access to a link to a questionnaire package. Through the online SurveyXact survey system students could complete the package at any time outside regular school hours. The system automatically adjusts the survey setup for computer screens, tablets and smart phones. This minimizes practical obstacles and increases feasibility and response rate.

### Data collection procedures

Quantitative data are collected at all four measure points (Fig. 1). In addition, fixed questions have been given to school staff, focusing on implementation issues. The semi-structured interviews will take place at 3 months follow up. Here 15 randomly selected students from the intervention schools will be invited, and the interviews depart from overall experiences of the HBI programme in terms of satisfaction, benefits and room for programme improvements.

### Statistical power and data analyses

The statistical power estimation was based on two comparison groups, α level = 0.05, and average within-cluster sample size of 70 students. In each group, 10 clusters are needed to achieve a statistical power of 81%. This is based on a meta-analysis [45] reporting a standardized weighted effect size (Cohen’s d) of 0.28 from 35 studies examining intervention effects on body images variables, and assuming a within-cluster dependency of no more than 3% (ICC = 0.03). The expectation of a rather low ICC is fair for variables related to psychological or mental health outcomes as selection factors like socioeconomic status variables affect these variables less than for example academic performance. The total required sample size thus becomes; 10 × 2 groups× 70 students in each cluster ~ 1400 students.

The outcome data will be analysed using mixed model regression due to several layers of dependency (i.e., correlated data) between students within schools and classes, and between the repeated data collected from the same student. These variables (schools, classes and initial measurements, or intercepts) will be included as separate random factors in order to correctly adjust the error bands. The restricted maximum likelihood procedure also handles missing data more flexibly by estimating unbiased parameter estimates using all the available data given a random missing mechanism may be assumed.

Transcribed qualitative interview data will be organized into QKS N’Vivo 10, and will be analysed according to the principles of systematic text condensation [46]. This involves 1) review of the data to get an overall impression; 2) identifying meaningful units representing different experiences 3) condense the significant units in subgroups and 4) synthesis and developing categories. Two researchers run the analysis separately, and then compare their findings until a point of unified understanding and consensus is reached. The Consolidated criteria for reporting qualitative research (COREQ) will be used to ensure high quality qualitative research [47].

### Timeline

The HBI programme was piloted March–April 2016. After minor adjustments, school principals were contacted from May–September 2016, and accepting schools were randomized in September. The intervention was conducted during October–December 2016, followed by a post-test in December 2016–January 2017, a 3 months- and 12-months follow-up in March–April and December 2017–January 2018 respectively (Fig. 1). Data files will be cleaned in February–March 2018, and the data analyses will start in March 2018.

## The intervention

### Framework

The HBI programme aims to change attitudes, believes and knowledge related to idealized lives and bodies, to combat the internalization of sociocultural ideas about body shape, as well as strengthen skills that will promote positive body image and prevent DE. It rests on *sociocultural theory* about how societal ideals of beauty are transmitted and internalized through a variety of channels such as family, peers, media, and that psychological development and learning emerges through interpersonal relations and actions with the social environment [48]. When internalizing such ideals, satisfaction or dissatisfaction with appearance will depend on to what extent individuals meet the sociocultural ideals. The programme also rests on the integrated etiological model of risk and protective factors [34, 42], and theories of embodiment [33] within the realm of positive psychology [49].

The intervention method is based on the *Elaboration Likelihood Model.* According to this model repeated exposure to a message facilitates cognitive elaboration of this message and increases the likelihood that the message is processed through a central, rather a peripheral cognitive route [50, 51]. In the HBI programme elaboration is facilitated by a high level of student activity around issues of common interest to them, i.e. how to promote a positive body experience and self-esteem and a healthy lifestyle. In addition, and in accordance with previous findings [20, 27, 28] elaboration is facilitated by the multiple session approach.

### Structure and content

The first and third authors, specialized in physical activity and health, sports nutrition, motivational interviewing, DE and BD among adolescents, conducted the programme. School teachers were allowed to be present in the classroom, however, without participating. To account for programme attendance, each student’s participation was registered at all intervention sessions. The intervention comprises three interactive workshops with a duration of 90 min each, i.e. two school hours. The three workshops were arranged in a classroom during regular school hours, and about 60 boys and girls (i.e. two school classes) participated. Three weeks interval between the workshops resulted in a 3 months intervention period.

Each workshop was adapted to suit adolescents 15–16 years of age with respect to their cognitive development and ability to abstract reasoning, and they comprised the main themes “body image”, “media literacy”, and “lifestyle”, respectively. Table 1 provides an overview of the programme content and targets. Parts of the school curriculum echo themes from the workshops, however without a comparable amount of focus, presentation methods, and learning techniques. As a result of the pilot study among 120 12th grade high schoolers only minor adjustments were made. Hence, some reiterated questionnaire items related to body perception and nutrition were deleted to reduce the risk of error variance due to acquiescence bias, and the amount of workshop assignments was reduced to allow for more time allocated to discuss mood and body satisfaction issues.

**Table 1:** Outline of content and targets of workshops #1 - #3 in the HBI programme

#1 Body image	
Main content	Targets
Project introduction	Experience of meaningfulness and motivation
Influencing factors on body perception. What promotes and reduces positive body image, and how can we enforce the health promoting factors?	Body image and body acceptance
Where does body idealization come from? Why does it conflict with positive body image, and potential health consequences from striving for the idealized body?	Psychoeducation to reduce idealization and internalization of a particular body ideal
Fat talk and focus on lifestyle only related to appearance in everyday communication. To what degree do we participate, how does it make us feel, and can we reduce it?	Reduce fat talk and negative body talk
Introduction to self-talk and self-esteem in WS#2	Stimulate motivation for next WS
#2 Media literacy	
Main content	Targets
Social media perception and use. Empower yourself to choose mood enhancing over mood destructive content	Enhance media literacy
Extreme exposure without filter equals need to be critical to sources of information and awareness of retouching	Enhance media literacy
The nature of comparison, how to recognize destructive comparison and reduce its presence in everyday life	Reduce amount of comparison
Strengthen acceptance and love for individual differences, defining characteristics of ones’ own and among friends. Students write down compliments to a friend and him/herself unrelated to appearance	Improve positive self-talk Improve self-compassion
Experiences and benefits of positive self-talk	Improve skills to strengthen self-esteem
#3 Lifestyle	
Main content	Targets
Benefits on body experience from listening to bodily needs such as physical activity and healthy eating	Improve experience of embodiment
Truths and myth about lifestyle products and literature	Improve ability to reject exercise and nutritional myths - health information literacy
From aesthetic to functional focus; how can change in focus improve body experience and healthy lifestyle that again benefit well-being?	Change from potential unhealthy focus to healthy focus on the body
How may regular exercise and smart nutrition promote positive body image and what are the basic recommendations?	Body experience enhancing attitudes and behaviours

#### Outcome measures and variables

The questionnaire package is outlined in Table 2. Apart from demographic questions this package covers the primary and secondary outcome measures as well as the moderator/mediator variables. Fixed questions to school staff and interview data (students) cover aspects of feasibility. Finally, all students responded to questions regarding demographics as well as academic achievements in their last semester report in the obligatory subjects, i.e. English, Math, Norwegian, and Physical education, respectively.

**Table 2:** Overview of the instruments used to evaluate the efficacy of the HBI programme

	Outcome measures	Content
Main outcome variables	Experience of Embodiment Scale [33]	Body image
	EDE-Q-11 [52]	Disordered eating
Secondary outcome variables	The body image acceptance and action scale [53]	Body image
	Sociocultural Attitudes Towards Appearance Questionnaire-4 (SATAQ-4) [54]	Body image
	Drive for Leanness Scale (DLS) [55]	Body image
	The KIDSCREEN-10 [56]	Health related quality of life
	Self-developed Physical activity level/habits questionnaire	Lifestyle behaviours
	Self-developed Food frequency questionnaire	Lifestyle behaviours
	The Bergen Insomnia Scale [57]	Lifestyle behaviours
	Hopkins Symptom Checklist-10 (SCL-10) [58]	Symptoms of anxiety and depression
	Self-developed Social media questionnaire (to be published)	Impression management, Body and appearance and looks, Literacy, Social capital, Social media addiction
Mediator and moderator variables	Frost Multidimensional Perfectionism Scale [59]	Perfectionism
	Rosenberg self-esteem [60]	Self-esteem
	The Self Compassion Scale-12 [61]	Self-compassion
	The Resilience Scale for Adolescents [62]	Mental health protective factors

## Discussion

The present study is one of the first to integrate a health promotion and a disease prevention approach, as well as integrating standardized outcome measures and experiential findings.

In contrast to many previous studies, adherence to the intervention will be presented, thus increasing the validity and credibility of findings. Importantly, themes included in the intervention programme can to some extent be placed under themes in the ordinary schools’ curricula. This creates a potential for increased feasibility, but it also creates a test of the programme effects. Skills that are taught through the workshops might need to mature over time. Hence, a 12-month follow up using the same outcome measures might make it possible to identify both immediate and long-term effects, and to what extent the participants experience that the programme has been useful in their daily life.

Moreover, the integrated health promotion and disease prevention perspective may offer the possibility of empirically evaluating the theoretical relationship between BD and a positive body image. Notably, it will be possible to differentiate between health promoting outcomes and outcome related to DE.

In contrast to most previous studies, the inclusion of mediator/moderator variables and our large sample size allows for sub-group analyses in order to identify those who might or might not benefit from the intervention. Including both genders may be a challenge as BD may be unevenly developed by the age of 15–16 years. However, all students can potentially benefit from healthier attitudes and practices in relation to their own body and to their social responsibilities as peers and family members [34]. Thus, sub-group analyses may also comprise possible gender and cultural differences.

The potential for the generalizability of findings seems satisfactory as the study sample representing both urban and rural parts of a large population area, and comprising both public and private schools.

Some limitations should be mentioned. First, a non-blinded procedure can lead to a potential expectancy bias for the researcher and the participating students in favour of the intervention. A related issue is the fact that those who implemented the HBI programme for practical reasons also interviewed participating students about how they experienced the programme. Secondly, underreporting may be the result of the programme format in which some students might have been reluctant to discuss personal and private issues in large classrooms and during the workshops when teachers were present. A related issue is whether the adjustment of questionnaire items to omit sensitive or unclear items is sufficient to prevent underreporting. Thirdly, completing a large questionnaire at four measure points may introduce the possibility of random responding due to an acquiescence bias, or some “learning effects”. The latter seems unlikely given the considerable time intervals between each measure point.

Despite these limitations, it is expected that the quantitative and qualitative evaluation of the BHI programme will merit larger scale dissemination efforts within the school health system, and possibly within relevant contexts in the primary health care services. Thus, apart from the customary publishing in international high-impact journals, the study’s purpose is to bridge the gap between research and practice. Thus, we aim to communicate findings to regional and national decision makers in the education and health care services.

## Abbreviations

BD: Body dissatisfaction
DE: Disordered eating
DLS: Drive for Leanness
ED: Eating disorder
EDE-Q: Eating Disorder Examination Questionnaire
HBI: Healthy Body Image
ICC: Intra-class correlation
SATAQ-4: Sociocultural Attitudes Towards Appearance Questionnaire
SCL: Symptom Checklist
WS: Workshops
